# Supplementary material for: Publics and UK parliamentarians underestimate the urgency of peaking global greenhouse gas emissions
Source: Commun Earth Environ. 2025 Oct 2;6(1):747. doi: 10.1038/s43247-025-02655-w (PMC12491064; doi:10.1038/s43247-025-02655-w)
Supplement: Supplementary file 2 — Reporting Summary [file 43247_2025_2655_MOESM2_ESM.pdf]

nature portfolio

Corresponding author(s): DAPR: COMMSENV-25-0005-T

Last updated by author(s): Jul 8, 2025

Reporting Summary

Nature Portfolio wishes to improve the reproducibility of the work that we publish. This form provides structure for consistency and transparency in reporting. For further information on Nature Portfolio policies, see our Editorial Policies and the Editorial Policy Checklist.

Statistics

For all statistical analyses, confirm that the following items are present in the figure legend, table legend, main text, or Methods section.

n/a	Confirmed	
☐	☒	The exact sample size (*n*) for each experimental group/condition, given as a discrete number and unit of measurement
☐	☒	A statement on whether measurements were taken from distinct samples or whether the same sample was measured repeatedly
☐	☒	The statistical test(s) used AND whether they are one- or two-sided<br>*Only common tests should be described solely by name; describe more complex techniques in the Methods section.*
☐	☒	A description of all covariates tested
☒	☐	A description of any assumptions or corrections, such as tests of normality and adjustment for multiple comparisons
☐	☒	A full description of the statistical parameters including central tendency (e.g. means) or other basic estimates (e.g. regression coefficient) AND variation (e.g. standard deviation) or associated estimates of uncertainty (e.g. confidence intervals)
☐	☒	For null hypothesis testing, the test statistic (e.g. *F*, *t*, *r*) with confidence intervals, effect sizes, degrees of freedom and *P* value noted<br>*Give P values as exact values whenever suitable.*
☒	☐	For Bayesian analysis, information on the choice of priors and Markov chain Monte Carlo settings
☒	☐	For hierarchical and complex designs, identification of the appropriate level for tests and full reporting of outcomes
☒	☐	Estimates of effect sizes (e.g. Cohen's *d*, Pearson's *r*), indicating how they were calculated

Our web collection on statistics for biologists contains articles on many of the points above.

Software and code

Policy information about availability of computer code

Data collection	No software was used for the data collection - the data was collected on our behalf by specialists companies
Data analysis	Custom data was used for this analyses. This is available on Code Ocean through the following link: https://doi.org/10.24433/CO.7717651.v1. Data was analysed using Stata v15.1

For manuscripts utilizing custom algorithms or software that are central to the research but not yet described in published literature, software must be made available to editors and reviewers. We strongly encourage code deposition in a community repository (e.g. GitHub). See the Nature Portfolio guidelines for submitting code & software for further information.

Data

Policy information about availability of data

All manuscripts must include a data availability statement. This statement should provide the following information, where applicable:

- Accession codes, unique identifiers, or web links for publicly available datasets
- A description of any restrictions on data availability
- For clinical datasets or third party data, please ensure that the statement adheres to our policy

Data and replication code for the publics analyses has been made available on Code Ocean. This can be accessed using the following url: https://doi.org/10.24433/CO.7717651.v1. The data for the MPs surveys is taken from aggregate data summary tables provided by the survey company.

Research involving human participants, their data, or biological material

Policy information about studies with human participants or human data. See also policy information about sex, gender (identity/presentation), and sexual orientation and race, ethnicity and racism.

Reporting on sex and gender	The unweighted balance for MPs in the data is 75% male to 25% female, in comparison to the then MP population of 66% men to 34% women. This data was provided by the survey company as background information, rather than asked as part of our questions in the omnibus survey.<br><br>In the publics surveys, individuals were asked what their sex was, and quotas were used to match the sex ratio present at the national level in each country.
Reporting on race, ethnicity, or other socially relevant groupings	Race or ethnicity variables were not used in our present study<br><br>For publics, the study asked individuals their subjective social status through the following question which is included as an independent variable in the regression analyses:<br>"In our society there are groups which tend to be towards the top and groups which tend to be toward the bottom. Below is a scale that runs top to bottom Where would you put yourself on this scale? (1. Bottom – 10. Top)."<br><br>For both publics and MPs, we draw on a measure of their party affiliation/preferences. The company for the MPs survey provided us with information on whether the MPs were Labour, Conservative, SNP or other. Given the sample sizes, we concentrate on differences between Labour and Conservative MPs (there were just 7 SNP respondents). For publics, they were asked which party they voted for at the 2019 UK General Election.
Population characteristics	See above
Recruitment	MP Survey:<br>Our questions were included in an omnibus survey of UK MPs carried out by Savanta. Further information on their methodology can be found here: https://savanta.com/audience-expertise/mps/<br><br>Publics surveys:<br>The sampling procedure for these surveys used quota sampling, with participants invited to participate through online panels. While these individuals opted in the receive survey invitations, such surveys have a strong track record at providing accurate results. As we refer to in the methods section, the survey company we used - Survation - were the most accurate in predicting the result of the 2017 UK General Election.
Ethics oversight	Ethics approval was provided by the University of East Anglia Faculty of Science Research Ethics Subcommittee for the MPs survey (Application ID: ETH2223-0766) and for the publics surveys (Application IDs: ETH2223-0575; ETH2324-0224)

Note that full information on the approval of the study protocol must also be provided in the manuscript.

Field-specific reporting

Please select the one below that is the best fit for your research. If you are not sure, read the appropriate sections before making your selection.

☐ Life sciences ☒ Behavioural & social sciences ☐ Ecological, evolutionary & environmental sciences

For a reference copy of the document with all sections, see nature.com/documents/nr-reporting-summary-flat.pdf

Behavioural & social sciences study design

All studies must disclose on these points even when the disclosure is negative.

Study description	This study utilizes a quantitative approach to examine knowledge of both MPs and members of the public of a key statement from the UN IPCC assessment reports.
Research sample	MPs were recruited through an MP panel run by Savanta, and fielded on one of their monthly omnibus surveys with a sample of 100 Members of Parliament representative by party, gender, age and region. The sample was chosen as the views of sitting MPs were of interest, and Savanta has an excellent record in providing high-quality samples from these.<br><br>The publics surveys were fielded to representative national samples in Great Britain, Germany, Chile and Canada. These countries were chosen to capture the views of publics in different continents (Europe, North America and South America). The data collection was undertaken by Survation who were chosen following a public tendering process.
Sampling strategy	MP Survey:<br>Our questions were included in an omnibus survey of UK MPs carried out by Savanta. Further information on their methodology can be found here: https://savanta.com/audience-expertise/mps/<br><br>Publics surveys:<br>The sampling procedure for these surveys used quota sampling, with participants invited to participate through online panels. The

	study aimed for a minimum of 1,500 respondents in Canada and Chile which would provide sufficient statistical power for regression analyses of the data, while the Great Britain and Germany samples aimed for a minimum of 2,000 respondents to enable a follow-up repeat survey of participants to take place 12 months later (and account for sample attrition in this second wave).
Data collection	MP Survey:<br>Our questions were included in an omnibus survey of UK MPs carried out by Savanta. Further information on their methodology can be found here: https://savanta.com/audience-expertise/mps/<br><br>The publics surveys were fielded by Survation, with participants recruited from online panels and taking the survey online.
Timing	MP survey:<br>6 September - 16 October 2023<br><br>Publics surveys:<br>Britain: 15 November - 18 December 2023<br>Canada: 14 – 30 November 2023<br>Chile: 14 -November – 4 December 2023<br>Germany: 20 November – 15 December 2023
Data exclusions	No data exclusion criteria were applied
Non-participation	Data collection continued up until all the required quotas had been met. Post-straification weights were applied afterwards to account for any imbalances.
Randomization	There was no randomization involved in this study

Reporting for specific materials, systems and methods

We require information from authors about some types of materials, experimental systems and methods used in many studies. Here, indicate whether each material, system or method listed is relevant to your study. If you are not sure if a list item applies to your research, read the appropriate section before selecting a response.

Materials & experimental systems

n/a	Involved in the study
☒	☐ Antibodies
☒	☐ Eukaryotic cell lines
☒	☐ Palaeontology and archaeology
☒	☐ Animals and other organisms
☒	☐ Clinical data
☒	☐ Dual use research of concern
☒	☐ Plants

Methods

n/a	Involved in the study
☒	☐ ChIP-seq
☒	☐ Flow cytometry
☒	☐ MRI-based neuroimaging

Plants

Seed stocks	*Report on the source of all seed stocks or other plant material used. If applicable, state the seed stock centre and catalogue number. If plant specimens were collected from the field, describe the collection location, date and sampling procedures.*
Novel plant genotypes	*Describe the methods by which all novel plant genotypes were produced. This includes those generated by transgenic approaches, gene editing, chemical/radiation-based mutagenesis and hybridization. For transgenic lines, describe the transformation method, the number of independent lines analyzed and the generation upon which experiments were performed. For gene-edited lines, describe the editor used, the endogenous sequence targeted for editing, the targeting guide RNA sequence (if applicable) and how the editor was applied.*
Authentication	*Describe any authentication procedures for each seed stock used or novel genotype generated. Describe any experiments used to assess the effect of a mutation and, where applicable, how potential secondary effects (e.g. second site T-DNA insertions, mosiacism, off-target gene editing) were examined.*
